# Occipital hairline changes in women with frontal fibrosing alopecia receiving low-dose oral minoxidil

**DOI:** 10.1016/j.jdin.2026.08.013

**Published:** 2026-09-11

**Authors:** Gabrielle Baker, Megan Thuy Han Nguyen, Rachel K. Greene, Milan Hirpara, Celine H. Phong, Natasha Mesinkovska

**Affiliations:** a Department of Dermatology, University of California, Irvine, California; b School of Medicine, California University of Science and Medicine, Colton, California

**Keywords:** frontal fibrosing alopecia, lichen planopilaris, minoxidil, scarring alopecia

*To the Editor:* Frontal fibrosing alopecia (FFA) is a scarring alopecia that can involve the occipital scalp, yet evidence-based treatment recommendations remain limited.^1^ Although low-dose oral minoxidil (LDOM) can improve eyebrow and scalp hair density in FFA, its role in occipital hairline improvement remains understudied.^2^^,^^3^ To quantify this association, we retrospectively compared occipital hairline changes in women with FFA who received LDOM to those who did not.

We identified 186 patients with FFA in a university alopecia registry (January 2022 to May 2025). Inclusion required standardized occipital hairline photographs at baseline and ≥5-month follow-up. Patients were divided into LDOM-treated and non-LDOM cohorts (Fig S1, available via Mendeley at https://data.mendeley.com/datasets/7jxm8dyfh8/2). Concomitant therapies were permitted if unchanged from baseline through follow-up; patients with medication changes were excluded. Three independent graders (NAM, MH, CP) blinded to treatment assignment and image chronology used the posterior hairline component of FFA Severity Index (FFASI), a validated 5-point FFA severity scale, to evaluate occipital margin hair loss.^4^ Interrater reliability was assessed using a 2-way random-effects intraclass correlation coefficient (ICC) for exact FFASI score agreement. Descriptive statistics and independent *t* tests were performed. To adjust for baseline FFASI severity differences, follow-up FFASI scores were assessed using analysis of covariance.

Thirty women met the inclusion criteria, 20 LDOM-treated (mean age: 64.6 ± 10.2 years) and 10 non-LDOM (mean age: 67.9 ± 10.4 years), with comparable disease duration (LDOM: 27.8 ± 29.5 months; non-LDOM: 33.9 ± 27.6 months; *P* = .59) and photographic follow-up (10.1 ± 4.4 months; non-LDOM: 9.6 ± 2.0 months; *P* = .73). Interrater reliability was excellent (ICC = 0.92; 95% CI [0.84, 0.97]). Baseline occipital FFASI severity differed between LDOM-treated and non-LDOM (2.7 ± 1.1 vs 1.8 ± 0.9; *P* = .034). The LDOM cohort received a mean dose of 2.3 ± 0.7 mg/day for 9.8 ± 4.3 months. Concomitant therapies were common (≥3 treatments in 50% LDOM vs 40% non-LDOM; Tables S1 and S2, available via Mendeley at https://data.mendeley.com/datasets/7jxm8dyfh8/2).

After adjusting for baseline severity, LDOM was associated with a 0.87-point lower follow-up occipital FFASI score (β = −0.87; 95% CI [−1.40, −0.34]; *P* = .002). Score decreases <1 reflect measurable differences in FFA severity as grades are defined by centimeter ranges of hairline recession (Fig 1).^4^ Improvement occurred in 75% of LDOM (*n* = 15) and 20% of non-LDOM (*n* = 2) patients.

**Fig 1:**
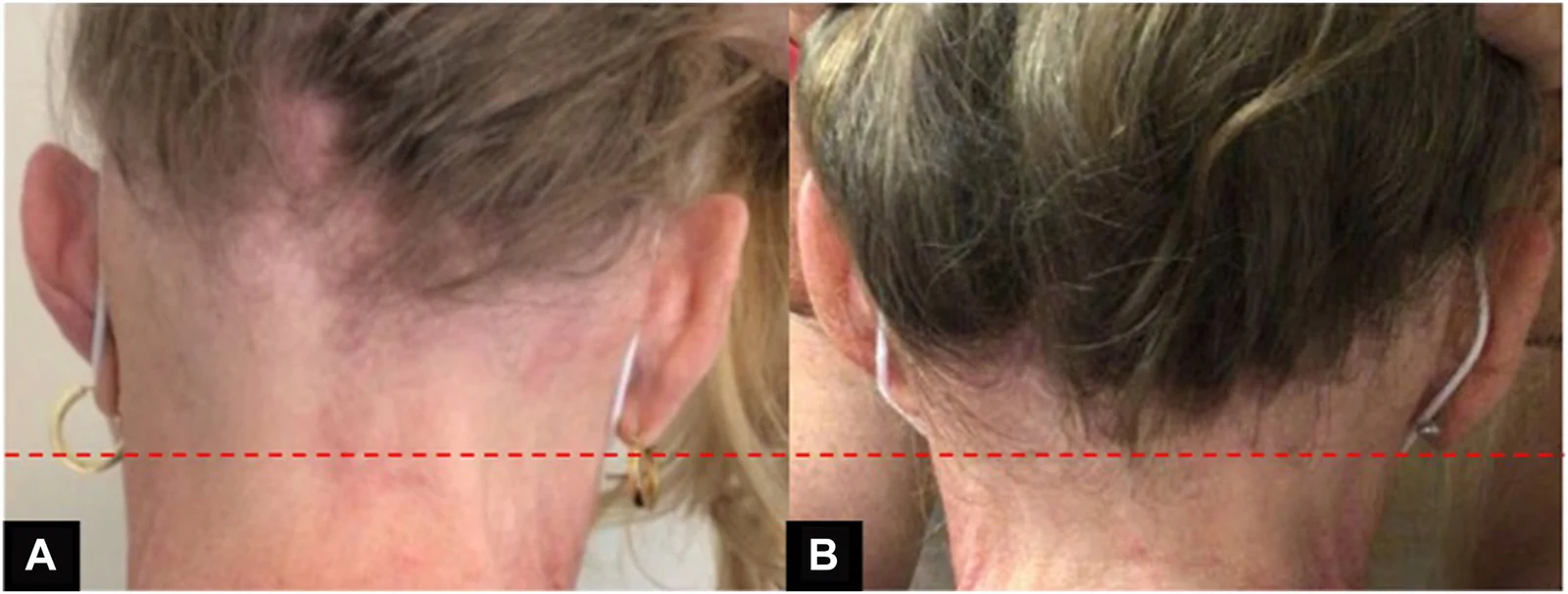
FFA patient demonstrating occipital hairline improvement with adjunctive LDOM 1.25 mg daily. FFASI 3-point decrease from baseline **(A)** to follow-up 6.6 months **(B)**. Concomitant therapies were maintained throughout follow-up: hydroxychloroquine 200 mg orally twice a day, intralesional triamcinolone 2.5 mg/mL, platelet-rich plasma (2 sessions), and topical minoxidil 5%, and was maintained throughout the study interval.

The biological basis for occipital FFASI score improvement remains uncertain. Prior studies suggest that the occipital FFA may retain vellus hair loss, perifollicular lymphocytes, lamellar fibrosis, and occasional terminal follicle preservation, which could partially explain the observed association.^1^ Additionally, oral minoxidil has previously demonstrated anti-inflammatory and antifibrotic effects.^5^ Although our study did not assess these mechanisms, they may offer biologic contextualization for the observed LDOM-associated occipital FFASI improvement and warrants further investigation.

Limitations included a small cohort, limiting generalizability. Although baseline FFASI differences were adjusted using analysis of covariance, concomitant therapy use, potential overlapping androgenic alopecia, and photographic variability may attenuate the observed treatment effects. Given our real-world retrospective study design, occipital FFASI improvement cannot be attributed to LDOM alone, nor can response predictors be inferred. Nonetheless, these exploratory findings suggest that adjunctive LDOM may be associated with occipital FFASI improvement in women. Prospective controlled monotherapy trials are needed to validate these observations.

### **Declaration of generative AI and AI-assisted technologies in the writing process**

During the preparation of this work the author(s) used ChatGPT in order to check spelling and grammar. After using this tool/service, the author(s) reviewed and edited the content as needed and take(s) full responsibility for the content of the publication.

## Conflicts of interest

None disclosed.
